# Ca^2+^-calmodulin inhibits tail-anchored protein insertion into the mammalian endoplasmic reticulum membrane

**DOI:** 10.1016/j.febslet.2011.10.008

**Published:** 2011-11-04

**Authors:** Sarah Haßdenteufel, Nico Schäuble, Patrizia Cassella, Pawel Leznicki, Anika Müller, Stephen High, Martin Jung, Richard Zimmermann

**Affiliations:** a Medical Biochemistry and Molecular Biology, Saarland University, 66421 Homburg, Germany; b National Research Council Institute for Neuroscience and Department of Medical Pharmacology, University of Milan, 20129 Milan, Italy; c Faculty of Life Sciences, University of Manchester, Manchester M13 9PT, UK

**Keywords:** Calcium, Calmodulin, Membrane insertion, Tail-anchored membrane proteins, Trifluoperazine, CaM, calmodulin, ER, endoplasmic reticulum, Get, guided entry of tail-anchored proteins, TA, tail-anchored, TRC, transmembrane recognition complex

## Abstract

Cytosolic components and pathways have been identified that are involved in inserting tail-anchored (TA) membrane proteins into the yeast or mammalian endoplasmic reticulum (ER) membrane. Searching for regulatory mechanisms of TA protein biogenesis, we found that Ca^2+^-calmodulin (CaM) inhibits the insertion of TA proteins into mammalian ER membranes and that this inhibition is prevented by trifluoperazine, a CaM antagonist that interferes with substrate binding of Ca^2+^-CaM. The effects of Ca^2+^-CaM on cytochrome b_5_ and Synaptobrevin 2 suggest a direct interaction between Ca^2+^-CaM and TA proteins. Thus, CaM appears to regulate TA insertion into the ER membrane in a Ca^2+^ dependent manner.

## Highlights

► We analyzed the effect of Ca^2+^-calmodulin on membrane integration of tail-anchored proteins. ► We found that Ca^2+^-calmodulin inhibits membrane insertion of TA proteins. ► This inhibition was prevented by trifluoperazine, a CaM antagonist. ► The effect of Ca^2+^-CaM was also seen for a purified TA protein. Ca^2+^-CaM can be cross-linked to TA proteins.

## 1 Introduction

The endoplasmic reticulum (ER) membrane is one of the major cellular sites for the membrane integration of nascent membrane proteins and newly synthesized tail-anchored (TA) membrane proteins, i.e. proteins that are membrane-embedded via a carboxy-terminal transmembrane domain [1], [2], [3]. TA proteins that are first inserted into the ER membrane are involved in a variety of biological processes, including signal sequence-dependent protein transport into the ER, ER calcium leakage, ER-associated protein degradation, apoptosis, and vesicular trafficking. Thus TA proteins may remain in the ER membrane or relocate to the nuclear envelope or to any of the membranes involved in endo- or exocytosis. In the last five years, pathways and components have been identified that are involved in this posttranslational membrane insertion in yeast [4], [5] and mammals [6], [7], [8], [9], [10], [11], [12], [13], [14]. Some TA proteins can enter the ER membrane unassisted, including mammalian cytochrome b_5_ (Cytb5) and the human protein tyrosine phosphatase 1B (PTP1B) [15], [16], [17]. Other TA proteins interact with SRP [6], cytosolic molecular chaperones [7], [10], or TA-dedicated machinery, such as the cytosolic transmembrane recognition complex (TRC) in mammals [8], [14] or the guided entry of tail-anchored proteins (Get) system, in yeast [4], [5], and involve an ER membrane resident receptor and/or TA-insertase (WRB in mammals, Get1/2 in yeast) [4], [18]. Human Synaptobrevin 2 (Syb2) was the first TA protein shown to demonstrate an energy requirement for membrane insertion [19]. It was further demonstrated that this energy requirement was due to the involvement of either SRP or TRC40/Asna-1. Human Sec61ß appears to be a TA protein that requires Hsp70 and Hsp40 chaperones, SRP, or TRC40/Asna-1 [6], [10]. In addition, both model proteins depend on an ER-resident membrane receptor or TA-insertase [19].

To address regulatory mechanisms of TA protein biogenesis, we analyzed whether the membrane insertion of several types of mammalian TA proteins was affected by Ca^2+^-CaM. Specifically, we studied Cytb5, Sec61ß, and Syb2 as model TA proteins. All three proteins were extended at their carboxyl termini via an opsin-derived 13- or 28-amino acid N-glycosylation site to create a reliable assay for membrane insertion. We found that TA insertion into the mammalian ER membrane was generally inhibited by Ca^2+^-CaM and that this inhibition was irrespective of the cytosolic pathway. Furthermore, we showed that the inhibition of TA insertion by Ca^2+^-CaM could be prevented by the CaM antagonist trifluoperazine (TFP) which interferes with CaM substrate binding. Based on our observations that Cytb5 membrane insertion is inhibited by Ca^2+^-CaM even in the absence of cytosolic factors and that Syb2 can be cross-linked to CaM in the presence of cytosolic factors we propose that Ca^2+^-CaM binds directly to the TA proteins.

## 2 Materials and methods

### 2.1 Materials

Rabbit reticulocyte lysate was obtained from Promega and [^35^S]methionine was from Perkin Elmer. Apyrase (grade VII from potato), CaM (from bovine testes) and TFP were purchased from Sigma. Proteinase K was from Roche and the cross-linking reagent was from Pierce. Chemicals for electrophoresis were purchased from Serva. PMSF and all other chemicals were obtained from Merck. Canine pancreatic micosomes were purified as described previously [20]. Glutathione-S-transferase (GST) and the fusion protein consisting of amino terminal GST linked to rat CaM were produced in *Escherichia coli* and purified according to standard procedure.

### 2.2 Protein transport experiments

Precursor polypeptides were synthesized in reticulocyte lysate in the presence of [^35^S]methionine for 30 min at 30 °C. The translation reaction contained EGTA at a concentration of approximately 400 μM. Synthesis was inhibited by incubation for 5 min at 30 °C in the presence of cycloheximide (100 μg/ml) and RNaseA (80 μg/ml). Buffer, rough microsomes (RM), or semi-permeabilized HeLa cells [21] were added and incubated for 30 min at 30 °C for the posttranslational transport experiments. Where indicated, apyrase, Ca^2+^, CaM, or TFP were present at final concentrations of 2 units per ml, 0.7 mM, 0.26 mg/ml, or 200 μM, respectively. All samples were analyzed by SDS–PAGE and phosphorimaging (Typhoon-Trio imaging system with Image Quant TL software 7.7; GE Healthcare). Where indicated, microsomes were re-isolated by centrifugation or subjected to carbonate extraction. For the latter, microsomes were re-isolated, resuspended in 100 mM sodium carbonate pH 11.0 and incubated for 30 min at 0 °C. Subsequently, carbonate extract was separated from the carbonate-resistant membrane pellet by centrifugation at 190,000×*g* for 20 min at 2 °C.

Alternatively, a fusion protein consisting of amino terminal GST linked to Cytb5 with the opsin-derived 28-amino acid N-glycosylation site was produced in *E. coli* and the Cytb5-ops moiety was released from the GST by thrombin cleavage as previously described [17]. Cytb5-ops was radiolabeled with [^14^C]formaldehyde according to standard procedures [20] and re-isolated by gelfiltration in phosphate buffer (100 mM sodium phosphate, pH 7.4). Transport assays were carried out in buffer (0.2 M sucrose, 50 mM KCl, 2 mM Mg acetate, 1 mM DTT, 20 mM HEPES–KOH, pH 7.5).

### 2.3 Analytical procedures

Cytb5 was synthesized and incubated as described above. Subsequently, the samples were divided into five aliquots and incubated with increasing concentrations of proteinase K for 60 min at 0 °C as indicated. After inhibition of the protease with 10 mM phenylmethylsulphonyl fluoride (PMSF), all samples were subjected to SDS–PAGE and phosphorimaging.

Syb2 was synthesized and incubated as described above. Subsequently, the samples were incubated with 4 mM 1-ethyl-3-[3-dimethylaminopropyl]carbodiimide hydrochloride (EDC) for 40 min at room temperature in 100 mM KCl, 0.1 mM CaCl_2_, 20 mM HEPES-KOH, pH 6 as described [22], [23]. All samples were analyzed by SDS–PAGE and phosphorimaging.

## 3 Results

### 3.1 Insertion of different model TA proteins into microsomes involves different cytosolic factors

**Fig. 1:**
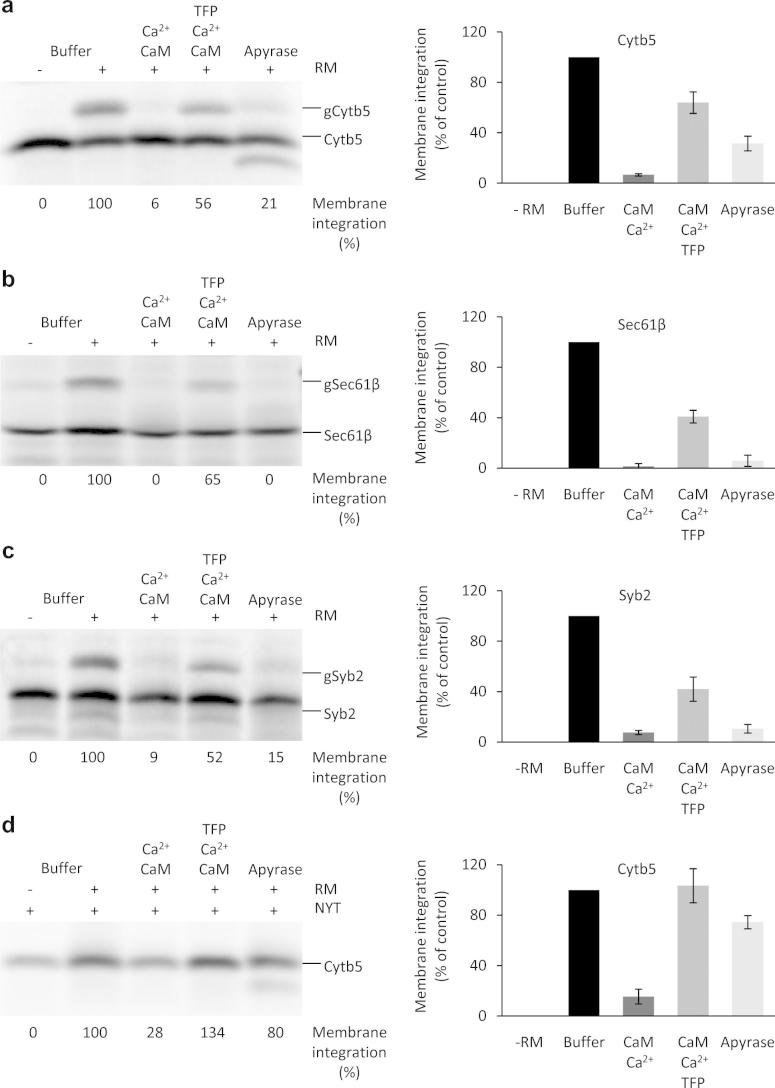

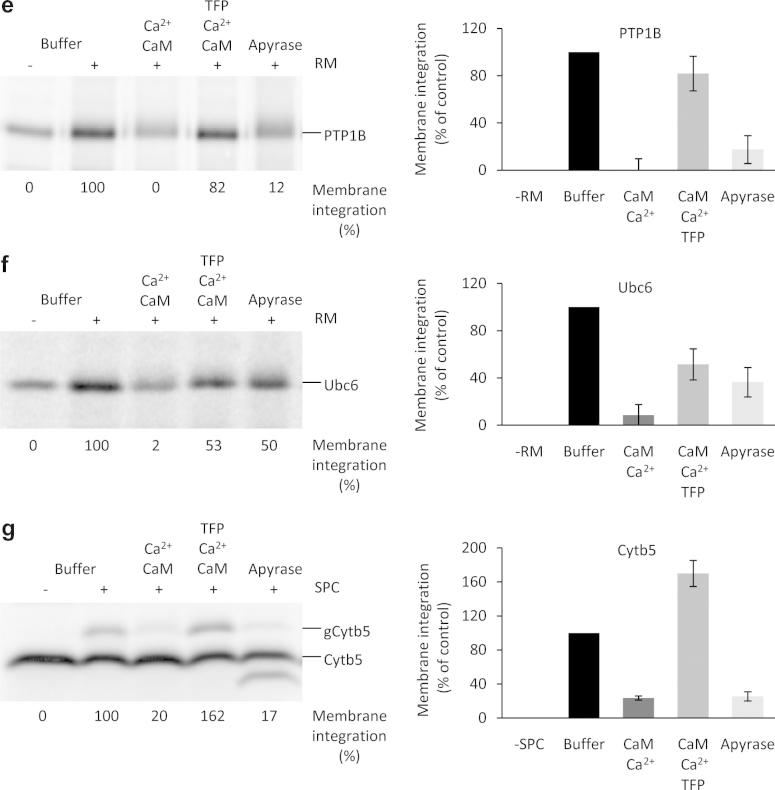
Insertion of model tail-anchored (TA) membrane proteins into rough microsomes is inhibited by Ca^2+^-CaM. TA proteins were synthesized in the presence of [^35^S]methionine. After inhibition of protein synthesis, the translation reactions were supplemented with buffer (lane 1) and canine pancreatic microsomes (RM) or semi-permeabilized HeLa cells (SPC) (lanes 2–5) and divided into aliquots. Where indicated, the aliquots were supplemented simultaneously with apyrase, Ca^2+^, CaM, and TFP. The experiment that is depicted in d was carried out in the presence of the acceptor tripeptide NYT to prevent N-glycosylation. After further incubation for 30 min, samples were left untreated (a,g) or subjected to either centrifugation (b,c) or carbonate extraction (d–f). Samples or sample pellets after centrifugation or carbonate extraction were subjected to SDS–PAGE and phosphorimaging. Only the areas of interest in single gels are shown in the left panels. The right panels show the mean values and the standard errors of the mean from at least four individual experiments. The amount of carbonate-resistant protein was corrected using the buffer background value. The efficiency of membrane integration for the buffer control (as assayed as either N-glycosylation or carbonate resistance) was set as 100%.

Before we investigated the effects of Ca^2+^-CaM on the insertion of mammalian TA proteins into the mammalian ER membrane, the energy dependence of insertion was confirmed for the model proteins. TA proteins were synthesized in the presence of [^35^S]methionine and the translation reactions were supplemented with pancreatic microsomes and divided into aliquots. Where indicated, the aliquots were supplemented with apyrase, which hydrolyzes ATP and ADP and leads to a depletion of GTP due to the action of nucleoside diphosphate kinases in the lysate. To allow membrane insertion, the aliquots were incubated further. One translation reaction was supplemented with buffer instead of microsomes and served as a negative control. Subsequently, all samples were subjected to SDS–PAGE and phosphorimaging analysis (Fig. 1a–f). As expected, there was no N-glycosylation in the absence of microsomes (Fig. 1a–f, lane 1 in the left panels), but there was membrane insertion as measured by glycosylation in the presence of microsomes for Cytb5, Sec61ß and Syb2 and as measured by carbonate resistance for Cytb5, PTP1B and Ubc6 (Fig. 1a–f, lane 2 in the left panels). However, when the translation reactions were depleted of nucleoside triphosphates, there was a significant reduction of glycosylation (Fig. 1a–f, compare lane 5 to lane 2, left panels). Thus, the in vitro insertion system proved suitable for the analysis of TA protein insertion into ER membranes and the known requirements for energy-dependent cytosolic factors were confirmed for the model proteins [10], [19]. As expected, the requirements for energy-dependent cytosolic factors varied for the different TA proteins (Sec61ß > Syb2 > PTP1B > Cytb5 > Ubc6).

### 3.2 Insertion of TA membrane proteins into the ER is inhibited by Ca^2+^-CaM

We showed previously that Ca^2+^-CaM can bind to the α-subunit of the Sec61 complex, thereby limiting Ca^2+^ leakage from the ER [24]. Binding of Ca^2+^-CaM to the Sec61 complex, however, does not interfere with signal peptide-dependent protein transport into the ER. Here, the question was whether Ca^2+^-CaM affects the Sec61-independent insertion of TA polypeptides into microsomal membranes. Posttranslational membrane insertion of TA proteins was carried out as described above. However, where indicated, the aliquots were supplemented with Ca^2+^-CaM (Fig. 1a–f). In all cases, the presence of Ca^2+^-CaM led to a significant reduction in glycosylation of the tail anchors (Fig. 1a–f, compare lane 3 to lane 2 in left panels). The inhibitory effect of Ca^2+^-CaM was more pronounced than the effect of energy depletion (Fig. 1a–f, compare lane 3 to lane 5). We note that this effect was not due to N-glycosylation inhibition *per se*, but was rather due to inhibition of membrane insertion. That is, the observed effect was similar to that seen when carbonate resistance was employed as an assay for membrane insertion (Cytb5, PTP1B, Ubc6) (Fig. 1d–f). Thus, the insertion of mammalian TA proteins into mammalian microsomes is sensitive to Ca^2+^-CaM and this sensitivity is irrespective of the cytosolic factors involved. Furthermore, Ca^2+^-CaM inhibited membrane insertion of Cytb5 when ER membranes derived from human cells in the form of semi-permeabilized cells were used instead of microsomes (Fig. 1g).

Next, we investigated whether the presence of a CaM-antagonist that interferes with substrate binding by CaM, such as TFP [25], interferes with the inhibitory effect of Ca^2+^-CaM on TA insertion into the ER. Posttranslational membrane insertion of TA proteins was carried out in the presence of Ca^2+^-CaM as before with the addition of TFP (Fig. 1a–g). For all of the model proteins, TFP partially relieved the inhibitory effect of Ca^2+^-CaM (Fig. 1a–g, compare lane 4 to lane 3 in all left panels). Thus, the observed inhibitory effect of Ca^2+^-CaM on TA protein biogenesis specifically involves the substrate binding-site of CaM.

We note that it is rather laborious to detect the effect of Ca^2+^-CaM. In particular, the rabbit reticulocyte lysate that is used for model protein synthesis contains CaM, Ca^2+^, and an excess of EGTA to quench the Ca^2+^ (about 400 μM EGTA). The concentrations of these compounds can vary from one batch of lysate to the next. Therefore, one can observe partial inhibition and relief of inhibition by TFP even without the addition of exogenous CaM (data not shown). In addition, high concentrations of TFP (>150 μM) are harmful to membranes in the absence of CaM at least in combination with reticulocyte lysate; however, the action of TFP requires a four-fold molar excess over CaM [25]. Furthermore, it has to be taken into account that CaM has four binding sites for Ca^2+^ and that all binding sites for TFP are active only in the presence of Ca^2+^ [25].

On first sight, the used CaM concentration of 0.26 mg/ml or approximately 15 μM appears to be rather high in comparison to the nM concentrations of in vitro translation products. However, the half-maximal effect of CaM on TA membrane insertion was observed at 7.5 μM (data not shown). For comparison, physiological concentrations of CaM vary between 3 and 30 μM [26] and a concentration of 30 μM CaM was used to mimic the stimulatory effect of recticulocyte lysate on nuclear protein import [27].

### 3.3 Ca^2+^-CaM directly affects TA proteins

**Fig. 2:**
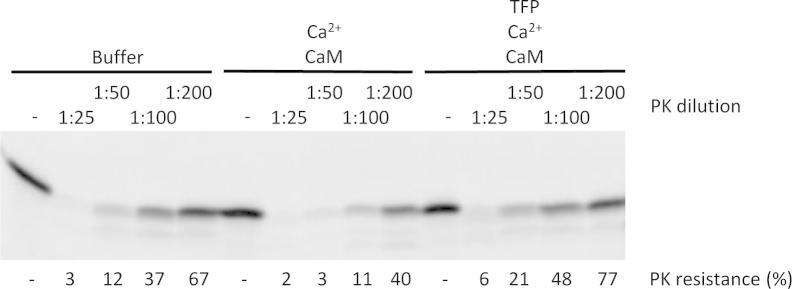
Protease accessibility of newly synthesized Cytb5 is altered by Ca^2+^-CaM. Cytb5 was synthesized in the presence of [^35^S]methionine and divided into three aliquots. The three translation reactions were supplemented with buffer, Ca^2+^-CaM, and TFP as indicated and incubated for 30 min at 30 °C. Subsequently, each of the three translation reactions was divided into five aliquots and incubated with increasing dilutions of proteinase K for 60 min at 0 °C as indicated (starting concentration 175 μg/ml). After protease inhibition, all samples were subjected to SDS–PAGE and phosphorimaging. Only the areas of interest of a single gel are shown. The experiment was carried out three times with similar results.

We next addressed the question of how Ca^2+^-CaM affects insertion of TA proteins into mammalian microsomes. Since both types of model TA proteins were affected, i.e. insertase-dependent and insertase-independent, irrespective of the cytosolic factors that aid insertion, it appeared that Ca^2+^-CaM could bind directly to TA proteins in solution. Three approaches were employed to address this question. First, differential protease sensitivity was used as a tool to detect differences in soluble Cytb5 in the presence and absence of Ca^2+^-CaM. Cytb5 was synthesized in the presence of [^35^S]methionine and the translation reactions were divided into several aliquots. The aliquots were supplemented and incubated with buffer, Ca^2+^-CaM, or Ca^2+^-CaM plus TFP. Subsequently, the aliquots were divided further and incubated with decreasing concentrations of protease. All samples were subjected to SDS–PAGE and phosphorimaging. Cytb5 was more sensitive to protease in the presence of Ca^2+^-CaM as shown by comparing the buffer samples with the Ca^2+^-CaM samples (Fig. 2). TFP prevented the increase in protease sensitivity of Cytb5 due to Ca^2+^-CaM. Thus, Ca^2+^-CaM either directly or indirectly affects the structure of Cytb5.

**Fig. 3:**
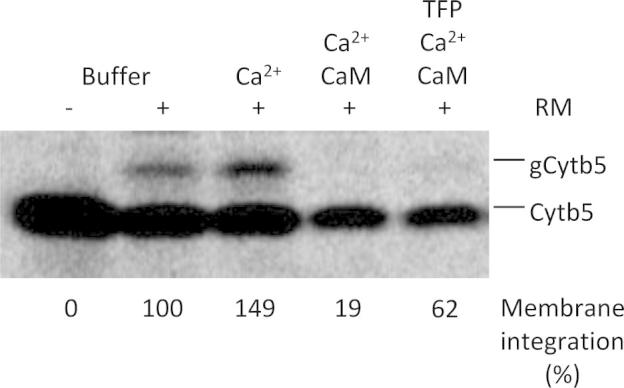
Insertion of purified radiolabeled Cytb5-ops into rough microsomes is inhibited by Ca^2+^-CaM. The experiment was performed in analogy to Fig. 1, except that purified Cytb5-ops rather than in vitro synthesized Cytb5 was employed and transport was carried out in buffer, i.e. in the absence of cytosolic factors. Only the areas of interest of a single gel are shown. The experiment was carried out three times with similar results.

Second, we asked if membrane insertion of purified Cytb5 was affected in the absence of cytosolic factors. Cytb5-ops was purified from *E. coli* and ^14^C-labeled by reductive methylation. The labeled protein was then incubated with microsomes in the absence or presence of Ca^2+^-CaM. Ca^2+^-CaM inhibited membrane insertion of Cytb5-ops even in the absence of reticulocyte lysate (Fig. 3, compare lane 3 to 4). This inhibition was partially prevented by TFP (Fig. 3, compare lane 4 to 5). This indicates that Ca^2+^-CaM binds directly to Cytb5.

**Fig. 4:**
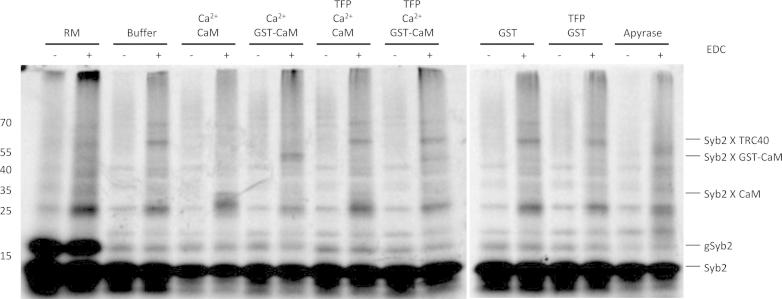
Binding of Syb2 to TRC40/Asna-1 is displaced by Ca^2+^-CaM. Syb2 was synthesized in the presence of [^35^S]methionine. The translation reaction was divided into aliquots, then supplemented and incubated with buffer, Ca^2+^-CaM, Ca^2+^-GST-CaM, GST, TFP, and apyrase as indicated. The final concentrations of GST and GST-CaM were 0.26 mg/ml. The aliquots were then incubated with the heterobifunctional cross-linking reagent EDC and all samples were subjected to SDS–PAGE and phosphorimaging. The positions of molecular mass standards are indicated (kDa). The experiment was carried out three times with similar results. We note that GST-CaM was equally efficient in inhibiting membrane insertion of TA proteins in the presence of Ca^2+^ as compared to CaM and that this inhibitory effect was suppressed by TFP (data not shown).

Third, we asked if Ca^2+^-CaM interfered with Syb2 binding to its dedicated cytosolic factor, TRC40/Asna-1. Syb2 was synthesized in reticulocyte lysate, i.e. in the presence of the cytosolic factors that facilitate its insertion into the membrane, and was then incubated with various agents before incubating with a zero-length cross-linking reagent. In the absence of any additional agents, one prominent cross-linking product was detected with an apparent mass of 65 kDa that most likely represents a cross-linking product between Syb2 and TRC40/Asna-1 (Fig. 4, lane 4). When Syb2 was incubated with microsomes to allow its membrane insertion, this cross-linking product was absent (Fig. 4, lane 2). Furthermore, the cross-linking product was absent after incubation with apyrase (Fig. 4, lane 18), The cross-linking product between Syb2 and TRC40/Asna-1was also absent after incubation with Ca^2+^-CaM or Ca^2+^-GST-CaM (Fig. 4, lanes 6 and 8), but was present when substrate binding of Ca^2+^-CaM was inhibited by TFP (Fig. 4, lanes 10 and 12). After incubation with Ca^2+^-CaM, however, a novel 35 kDa cross-linking product of Syb2 was detected (Fig. 4, lane 6). This product represents a cross-link between Syb2 (14 kDa) and CaM (17 kDa) since it was shifted to an apparent mass of about 55 kDa by employing GST-CaM (31 kDa) instead of CaM (Fig. 4, compare lane 6 to 8) and was absent after incubation with TFP or GST (Fig. 4, lanes 10, 12, 14 and 16). Thus, binding of Ca^2+^-CaM to Syb2 must displace cytosolic factors, such as TRC40/Asna-1, most likely by binding to the tail anchor.

## 4 Discussion

In this study we investigated whether membrane insertion of different types of mammalian TA proteins was affected by Ca^2+^-CaM. Specifically, we studied five model TA proteins that varied in terms of the auxiliary factors needed for membrane insertion: Cytb5 which involves but does not depend on cytosolic chaperones and which does not involve membrane insertase; Sec61ß which involves either chaperones, TRC40/Asna-1, or SRP plus a membrane-resident insertase; and Syb2 which involves TRC40/Asna-1 or SRP plus a membrane-resident insertase. We showed that TA protein insertion into the ER membrane was generally inhibited by Ca^2+^-CaM irrespective of the insertion pathway. Furthermore, we showed that the inhibition of TA insertion by CaM could be prevented by the CaM antagonist TFP which interferes with substrate binding by CaM. Based on TA insertion in the presence and absence of Ca^2+^-CaM in the absence of cytosolic factors and on chemical cross-linking of TA proteins in the presence of cytosolic factors, we propose that Ca^2+^-CaM binds directly to the tail anchors. Such binding is not entirely unexpected since Ca^2+^-CaM has been shown previously to bind to amphiphilic peptides in general [28] and to degradation products derived from signal peptides in particular [29]. It is also notable that TA protein biogenesis is regulated by the redox state of the cytosol in both yeast and mammals [9].

We propose that Ca^2+^-CaM can halt TA protein biogenesis prior to TA insertion into the ER membrane. For at least one TA protein, it must be important to prevent membrane insertion while CaM is saturated with Ca^2+^. We assume that such a TA protein is regulated by either Ca^2+^ or Ca^2+^-CaM in its membrane-resident form. Candidate proteins include TA proteins that are involved in vesicular transport (Syb2, VAMP2, and syntaxin), or apoptosis and autophagy (Bcl-2) [30], [31], [32]. Alternatively, binding of Ca^2+^-CaM may function to protect TA proteins from degradation prior to membrane integration. Future studies should address whether the inhibition of TA membrane insertion is limited to insertion into the ER membrane or whether insertion of TA proteins into the mitochondrial outer membrane can be inhibited in a similar way [33], [34].
